# Resilience-Enhancing Programs for Nurses in the Era of COVID-19: A Systematic Review and Meta-Analysis

**DOI:** 10.3390/healthcare14070906

**Published:** 2026-03-31

**Authors:** Wonjung Noh, Young Ko

**Affiliations:** College of Nursing, Gachon University, Incheon 21936, Republic of Korea; wjnoh@gachon.ac.kr

**Keywords:** COVID-19, nurses, post-pandemic, resilience, systematic review

## Abstract

**Highlights:**

**What are the main findings?**

- A systematic review and meta-analysis of six studies demonstrated that resilience-enhancing programs had a statistically significant positive effect on nurses’ resilience in the post-pandemic era.
- Despite the overall positive effect, substantial heterogeneity and low-to-very-low certainty of evidence indicate that the magnitude and consistency of the effect should be interpreted cautiously.

**What are the implications of the main findings?**

- Resilience-enhancing programs may support nurses’ psychological well-being in post-pandemic healthcare settings. However, additional high-quality, adequately powered trials are needed to strengthen the evidence base.
- Future studies should focus on resilience as the primary outcome and explore organizational-level outcomes.

**Abstract:**

**Background/Objectives**: In the post-pandemic era, growing concern about the mental health of healthcare professionals has led to the development of various resilience-enhancing programs. Although such programs are not new, having been implemented before the pandemic, it is important to investigate how post-pandemic programs differ from earlier ones. This review aimed to analyze resilience-enhancing programs for nurses and evaluate their effectiveness. **Methods**: This systematic review and meta-analysis adhered to the Preferred Reporting Items for Systematic Reviews and Meta-Analyses (PRISMA) guidelines. A search was performed in the Cochrane Library, the Cumulative Index to Nursing and Allied Health Literature (CINAHL), PubMed, and EMBASE. Six studies met the inclusion criteria. The meta-analysis was conducted using Stata version 16.0 (StataCorp LLC., College Station, TX, USA). **Results**: Six studies were included in the systematic review and meta-analysis. The characteristics of the included studies, such as country, study design, setting, population, outcome variables, and resilience-enhancing programs for nurses, were analyzed. The random-effects meta-analysis indicated a statistically significant positive effect on nurses’ resilience (SMD = 0.58, 95% CI 0.10 to 1.07, Z = 2.35, *p* = 0.019). **Conclusions**: This study provides foundational evidence for understanding resilience-enhancing programs for nurses and highlights their potential value in post-pandemic healthcare settings.

## 1. Introduction

During the coronavirus disease 2019 (COVID-19) pandemic, healthcare providers faced difficulties unprecedented in routine practice environments [1]. These included limited knowledge about diseases; shortage of personal protective equipment; and physical and psychological concerns such as burnout, anxiety, and sleep disturbances [2,3]. The COVID-19 pandemic, in particular, has been characterized as a traumatic event comparable to natural disasters, large-scale catastrophes, or serious disease outbreaks, with long-term negative effects on the mental health of healthcare workers [4]. A systematic review conducted during the pandemic reported a significant increase in post-traumatic stress disorder (PTSD) symptoms among frontline nurses, underscoring the substantial psychological burden experienced by this population [5].

In response to these cumulative stressors, some healthcare professionals chose to leave the profession altogether [6]. Consequently, the ability to confront adversity, adapt to challenging circumstances, and effectively manage problems has become increasingly important. Resilience has thus emerged as a key psychological resource.

Resilience is a dynamic process of positive adaptation in the face of significant adversity or stress [7]. Nurses who maintain continuous close contact with patients and are routinely exposed to complex and demanding healthcare environments are particularly vulnerable to occupational stress and, therefore, require greater resilience [8]. Higher levels of resilience among nurses are associated with reduced stress, burnout, fatigue, anxiety, and depression, as well as increased job satisfaction and retention [9].

Beyond buffering negative psychological outcomes, resilience is also closely linked to post-traumatic growth [10]. Post-traumatic growth refers to positive psychological changes that occur as individuals struggle with and make meaning of traumatic or highly stressful experiences, resulting in enhanced self-perception, deeper interpersonal relationships, and a reappraisal of life priorities [11]. Emerging evidence suggests that resilience not only mitigates the adverse effects of trauma but also facilitates post-traumatic growth by enabling individuals to interpret traumatic experiences more flexibly and engage in active coping strategies [12]. Several studies have reported post-traumatic growth among nurses following the COVID-19 pandemic, including increased personal strength, redefined life values, and improved relational connectedness [13,14]. These findings imply that resilience may function as a critical mediating factor in the transition from adaptation to psychological growth following traumatic experiences.

From an organizational perspective, nurse managers have increasingly sought strategies to enhance resilience and maintain workforce stability and quality of care [15]. Accordingly, various resilience-enhancing intervention programs have been developed and tested for nurses [16]. However, their effectiveness remains unclear. While some studies report significant improvements in resilience-related outcomes [17], others have found no meaningful effects [18]. Outcomes such as stress [19] and burnout [20] have been frequently examined; however, the findings remain heterogeneous.

Several quantitative and qualitative reviews have examined resilience interventions among healthcare professionals and students. However, many such reviews do not adequately reflect the unique context of the COVID-19 pandemic or the specific characteristics of interventions implemented during this period [1,16]. Given the profound and enduring impact of the pandemic on nurses’ psychological well-being, a comprehensive synthesis of resilience-enhancing interventions that explicitly considers post-pandemic conditions is clearly needed.

Therefore, to inform the development of effective resilience-enhancing programs for nurses in the COVID-19 era, it is essential to systematically analyze existing intervention programs and evaluate their effectiveness, defined as the extent to which nurses’ resilience improves. Accordingly, this study aims to conduct a systematic review of the core components, duration, and outcome variables of resilience-enhancing programs for nurses, thereby providing evidence-based guidance for future intervention development and implementation.

### Aims

This review aims to analyze the characteristics of resilience-enhancing programs for nurses and estimate their effectiveness, with resilience as the primary outcome of interest.

## 2. Materials and Methods

### 2.1. Design

This systematic review evaluated the effect of resilience enhancing programs for nurses. This study was conducted in accordance with the Preferred Reporting Items for Systematic Reviews and Meta-Analyses (PRISMA) guidelines [21]. The protocol for this systematic review was registered with the International Prospective Register of Systematic Reviews (PROSPERO) under registration number CRD 42023465013.

### 2.2. Search Strategy and Selection

Published articles were systematically searched in the following databases: the Cumulative Index to Nursing and Allied Health Literature (CINAHL; EBSCOhost), the Cochrane Library, Embase (Elsevier), and MEDLINE (PubMed).

The search strategy was developed using Medical Subject Headings (MeSH) and keywords based on the Participants, Intervention, Comparison, Outcome, and Study design (PICOS) framework, along with supplementary search terms (Supplementary Materials File S1). The population (P) included nurses exposed to occupational stressors, particularly those related to COVID-19. The interventions (I) of interest were structured resilience-enhancing programs designed to improve resilience, coping capacity, or related outcomes. Eligible comparison (C) conditions included no intervention, usual care, or alternative interventions. The primary outcome (O) was quantitative measures of resilience. Study designs (S) were limited to randomized controlled trials that provided sufficient statistical information to calculate effect sizes.

The search strategy included MeSH terms and keywords such as “nurses”, “education”, “training”, “intervention”, “program”, “resilience”, and “randomized controlled trial”. The primary search was structured around the PICOS framework, with supplementary terms such as “pandemic” and “COVID-19” included to enhance relevance. Only studies published in English and Korean within the past 10 years were considered eligible.

Inclusion and exclusion criteria were established a priori to ensure methodological rigor. Studies were included if they: (1) involved nurses exposed to occupational stressors, including those related to COVID-19; (2) evaluated a structured intervention explicitly designed to enhance resilience; (3) used random assignment to allocate participants to intervention and control groups; (4) reported quantitative outcomes measuring resilience; and (5) provided sufficient statistical data to calculate effect sizes.

Studies were excluded if they: (1) did not use random assignment; (2) evaluated interventions without a clearly defined resilience component; (3) lacked a control or comparison group; (4) did not report extractable quantitative data; or (5) were reviews, qualitative studies, conference abstracts, or non-peer-reviewed publications.

Study selection followed PRISMA guidelines [21]. Titles and abstracts were independently screened by two reviewers, followed by full-text assessment of potentially eligible articles. Disagreements were resolved through discussion until consensus was reached.

### 2.3. Search Outcomes

**Figure 1:**
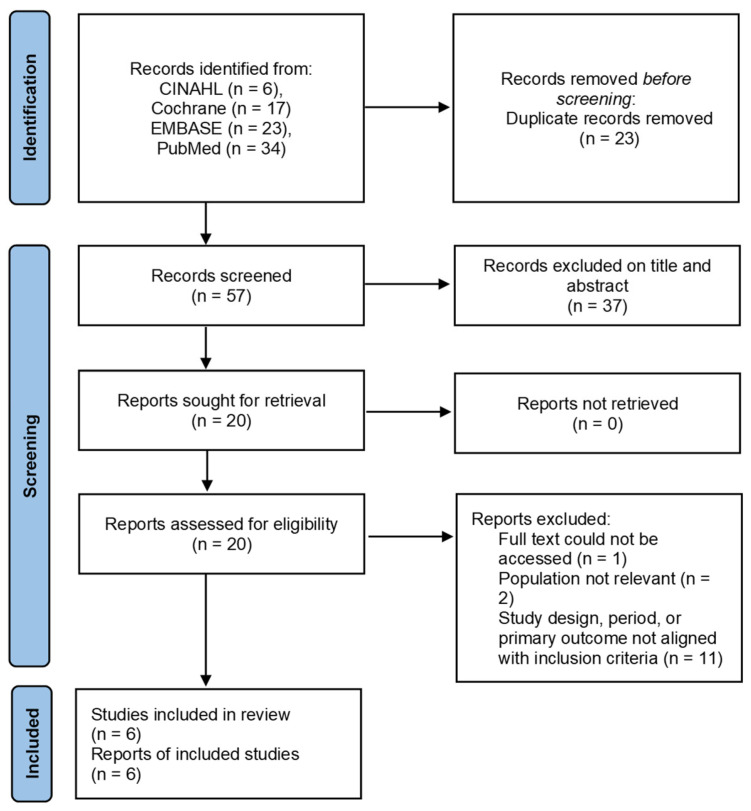
PRISMA flowchart of article selection.

A total of 80 records were identified through searches of the four databases. After removing 23 duplicate records, 57 articles were screened. Following title and abstract screening, 37 articles were excluded, and 20 full-text articles were assessed for eligibility. Finally, six studies met the inclusion criteria and were, therefore, included in this review and meta-analysis. The number of studies identified, screened, excluded, and included is summarized in the PRISMA 2020 flow diagram (Figure 1).

### 2.4. Quality Appraisal

**Figure 2:**
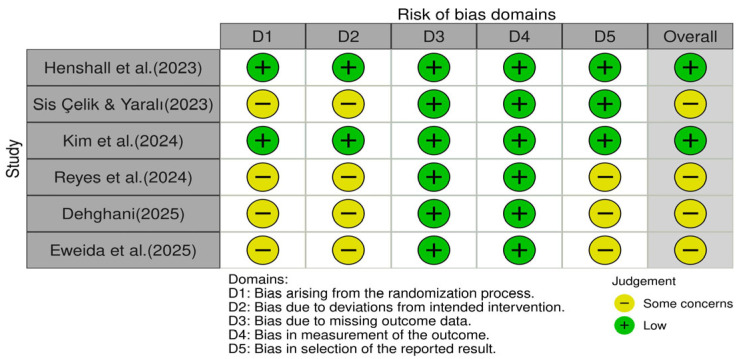
Quality appraisal of the studies included [23,24,25,26,27,28].

The methodological quality and risk of bias of the included studies were independently assessed by two reviewers using standardized tools [21] appropriate for each study design. Any discrepancies in the quality ratings were resolved through discussion until a consensus was reached. The methodological rigor of each study was evaluated across multiple domains, including participant selection, intervention description, and outcome assessment. The results of the quality appraisal are shown in Figure 2. The quality of the six included studies was assessed using the Cochrane risk-of-bias 2 (RoB 2) tool [22]. The assessments covered five domains: the randomization process (D1), deviation from intended intervention (D2), missing outcome data (D3), outcome measurement (D4), and selection of reported results (D5). Two studies (33.3%) were rated low risk, and four (66.7%) were rated as being of some concern (Figure 2). Although some risks of bias were identified (e.g., limited blinding and unclear allocation concealment), the results were interpreted with caution.

The Grading of Recommendations Assessment, Development, and Evaluation (GRADE) approach was used to assess the quality of evidence for each key outcome [23]. Table 1 presents the quality of evidence for resilience-related outcomes across the six included studies [24,25,26,27,28,29]. Randomized controlled trials (RCTs) were initially rated as high-certainty evidence but were downgraded due to serious risk of bias (primarily related to lack of blinding and reliance on self-reported outcomes) and serious imprecision (e.g., small sample sizes, pilot designs, and limited follow-up), resulting in an overall low certainty of evidence for resilience outcomes [24,25,26,27]. Quasi-experimental studies were initially rated as low-certainty evidence and were further downgraded due to serious risk of bias and imprecision, resulting in very low-certainty evidence [28,29].

Overall, although several interventions demonstrated statistically significant improvements in resilience [25,27,28,29], the certainty of the evidence remains limited, indicating that the true effect may differ substantially from the observed estimates.

### 2.5. Data Extraction and Synthesis

Data were extracted using a standardized data-extraction form that included key study characteristics (author, publication year, country, study design, setting, population, and outcome variables; Table 2). Two reviewers independently extracted the data. Discrepancies were resolved through discussion until a consensus was reached. When necessary data were not directly reported, effect sizes were calculated using the available statistical information. Studies that did not provide sufficient data for effect size calculations were excluded. To summarize the structure of the interventions, another standardized form was used to record details, such as the program name, protocol, theoretical framework, key components, and teaching methods (Table 3). For the meta-analysis, the sample size, pre- and post-intervention means, and standard deviations were extracted. All extracted data were cross-checked for accuracy and completeness by two independent reviewers.

### 2.6. Data Analysis

Random-effects meta-analyses and meta-regressions were performed using Stata version 16.0 (StataCorp LLC, College Station, TX, USA). A random-effects model using the DerSimonian–Laird method was applied to estimate the pooled effect size and account for heterogeneity among the studies. The standardized mean differences (SMDs) were calculated for resilience outcomes between the intervention and control groups using pre- and post-test results, and pooled mean effect sizes were presented with corresponding 95% confidence intervals (CIs). Statistical heterogeneity was assessed using the chi-square (χ^2^) test and the I^2^ statistic, with I^2^ values of 25%, 50%, and 75% representing low, moderate, and high heterogeneity, respectively. When significant heterogeneity was detected, meta-regression analyses were conducted to explore potential moderating variables, such as the theoretical framework, intervention duration, and intervention content. Publication bias was evaluated through visual inspection of funnel plots and Egger’s linear regression test. A sensitivity analysis was also performed. All statistical tests were two-tailed, and a *p*-value < 0.05 was considered statistically significant.

## 3. Results

### 3.1. General Characteristics

The general characteristics of the included studies are presented in Table 2. Since the COVID-19 pandemic, resilience intervention studies have been conducted in various countries, including South Korea, Iran, the United Kingdom, and the United States. Five studies provided interventions to clinical nurses and recruited participants online or offline using convenience sampling. While some studies have targeted nurses at high risk of PTSD [26,27], others have studied nurses without pre-existing mental health problems [25,28].

### 3.2. Resilience-Enhancing Programs for Nurses

Information on the programs used to enhance resilience is presented in Table 3. Across the six interventions, certain common themes emerged in the programs’ focus on enhancing nurses’ emotional regulation, cognitive flexibility, mindfulness, and positive affect. These elements represent key components of resilience-enhancing interventions. The Resilience Enhancement Online Program for Nurses (REsOluTioN) [24], Mental Health First Aid (MHFA) [29], Trauma Recovery Nursing Program [26], and mindfulness- and acceptance-based smartphone intervention [27] commonly emphasized emotion appraisal, the reframing of negative cognitions, reflective and critical thinking, and the cultivation of emotional support, indicating a predominantly cognitive–emotional orientation. These programs were grounded in structured theoretical frameworks, including cognitive–behavioral approaches (e.g., cognitive behavioral therapy [CBT] and acceptance and commitment therapy [ACT]) as well as Swanson’s caring theory, to promote psychological stability and adaptive coping. In contrast, the laughter yoga intervention [25] and the gratitude-based program [28] employed positive psychology and embodied affective techniques, using breathing exercises, laughter activities, or daily gratitude practices to induce relaxation and strengthen positive emotions.

In terms of delivery strategies, all six interventions emphasized online, flexible, and accessible formats, reflecting the need to accommodate nurses’ demanding schedules and shift-based work patterns. REsOluTioN [24], MHFA [29], and the laughter yoga program [25] primarily adopted synchronous group-based approaches, fostering interaction, social support, and collective engagement. REsOluTioN [24] further incorporated a blended structure of large-group sessions, preparatory learning, and small-group mentoring to enhance participant involvement. Conversely, the Trauma Recovery Nursing Program [26], mindfulness- and acceptance-based smartphone intervention [27], and gratitude training program [28] used asynchronous, individually paced modalities, such as URL-based sessions, app-delivered mindfulness exercises, or daily WhatsApp activities, thereby maximizing autonomy and feasibility. 

### 3.3. Publication Bias and Sensitivity Analysis

Publication bias was assessed using funnel plots and Egger’s regression test. The funnel plot showed a generally symmetric distribution of effect sizes around the pooled estimate, with no visual indication of small-study effects or directional asymmetry. This interpretation was supported by Egger’s regression test, which showed no statistically significant evidence of publication bias (*p* = 0.448), suggesting that selective reporting was unlikely to have influenced the meta-analytic results.

The robustness of the pooled effect was further evaluated using a leave-one-out sensitivity analysis. Sequential removal of each study produced pooled estimates that remained consistent in both direction and magnitude, with only minor fluctuations. These findings indicate that no single study exerted undue influence on the overall effect size and that the meta-analysis results are stable and reliable.

### 3.4. Effectiveness of Resilience-Enhancing Programs for Nurses

**Figure 3:**
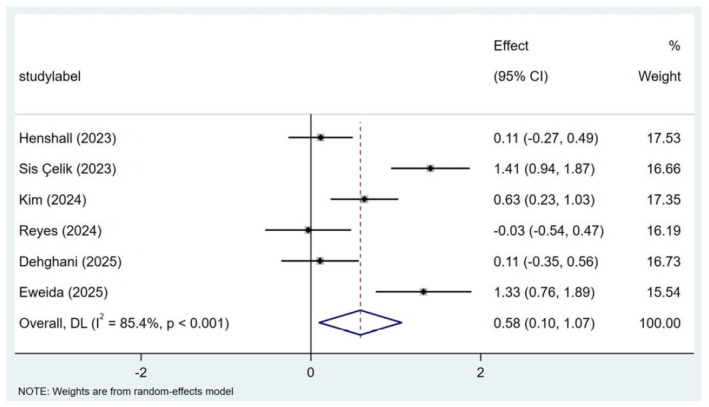
Meta-Analysis of Effectiveness of resilience-enhancing programs for nurses [24,25,26,27,28,29].

A random-effects meta-analysis showed that resilience-enhancing interventions had a statistically significant positive effect on nurses’ resilience (SMD = 0.58, 95% CI: 0.10–1.07, *p* = 0.019) (Figure 3). Substantial heterogeneity was observed across studies (I^2^ = 85.4%, Q(5) = 34.34, *p* < 0.001), indicating considerable variability in intervention effects. To explore potential sources of this heterogeneity, meta-regression analyses were conducted using three pre-specified moderators: intervention duration (≤4 weeks vs. >4 weeks), the presence of an explicitly stated theoretical framework, and inclusion of PTSD-related content in the intervention. None of these moderators significantly predicted the effect size (*p* > 0.05), and, therefore, none explained the observed between-study variability.

Consistent with these findings, subgroup analyses showed no statistically meaningful differences in intervention effects based on duration, theoretical grounding, or PTSD-related components, indicating that these factors did not account for the substantial heterogeneity across studies.

## 4. Discussion

This systematic review and meta-analysis aimed to examine resilience-enhancing programs for nurses and evaluate their effectiveness. Six studies were included. The random-effects meta-analysis demonstrated a statistically significant positive effect on nurses’ resilience; however, substantial heterogeneity was observed, and the certainty of the evidence ranged from low to very low. Therefore, the findings should be interpreted with caution.

To examine potential contributors to this variability, meta-regression analyses were conducted using three prespecified moderators: intervention duration, the presence of an explicitly stated theoretical framework, and the inclusion of PTSD-related content. None of these factors significantly predicted the effect size. Given the limited number of studies and variability in study characteristics, the analysis may have had insufficient power to detect moderating effects.

To better interpret the observed intervention effects, it is important to consider the conceptual and theoretical frameworks underlying these studies. Although the interventions varied in content, theoretical grounding, delivery modes, and participant characteristics, several conceptual commonalities emerged. Most programs emphasized core psychosocial processes associated with resilience, including emotional regulation, cognitive flexibility, mindfulness, and positive affect. Consistent with pre-pandemic resilience research [16], these interventions targeted protective processes that buffer the psychological impact of persistent occupational stressors in healthcare settings. This shared conceptual orientation is reflected in the structure and key components of the programs included in this review. For example, REsOluTioN [24], MHFA [29], the Trauma Recovery Nursing Program [26], and mindfulness- and acceptance-based smartphone interventions [27] promote adaptive coping through cognitive–emotional mechanisms grounded in CBT, ACT, and Swanson’s caring theory. In contrast, laughter yoga [25] and gratitude-based programs [28] draw on positive psychology-informed and embodied approaches to foster positive emotional states. Despite differences in theoretical traditions, these interventions appear to engage similar resilience-related processes through different pathways. Across programs, the proximal aim was to strengthen emotional well-being and reduce stress, consistent with short-term benefits reported in prior meta-analytic research.

Beyond these theoretical differences, temporal context may also have influenced the intervention focus. While pre-COVID-19 studies have addressed resilience in the context of chronic occupational stress and burnout [24], several studies conducted during the COVID-19 pandemic [26,27] shifted toward psychological recovery following large-scale traumatic exposure. These interventions addressed crisis-related phenomena, including trauma exposure, collective fatigue, moral injury, and organizational strain. The Trauma Recovery Nursing Program study [26], which targeted nurses at high risk for post-traumatic stress disorder, did not demonstrate a statistically significant improvement in posttraumatic symptoms or resilience. This finding should be interpreted cautiously, as baseline differences and sample characteristics may have influenced the results. In contrast, mindfulness- and acceptance-based smartphone interventions [27] reduced PTSD symptoms without a corresponding improvement in resilience. This difference may reflect variations in baseline symptom severity. Although these findings may be interpreted in relation to trauma-informed recovery models, they remain exploratory. Trauma theory conceptualizes adaptation as a staged process, progressing from stabilization to functional recovery and potential post-traumatic growth [30]. However, because staged recovery trajectories were not assessed, the relevance of this framework to the present findings remains conceptual.

Although several interventions were associated with reduced PTSD symptoms, improvements in resilience were less consistent. Given the relatively short intervention duration and predominantly online delivery formats, intervention intensity may have influenced resilience outcomes. A closer comparison of specific interventions implemented in high-risk settings further illustrates the potential role of contextual sensitivity. Among studies targeting nurses working in COVID-19 isolation wards [24,29], differences in resilience outcomes were observed despite similar risk profiles. The CBT-based MHFA intervention [29], which explicitly addressed nurses’ COVID-19-related experiences and incorporated structured social support, differed in focus from the online mentoring format of the REsOluTioN program [26]. While contextual tailoring and structured crisis-response elements may have contributed to these differences, the limited number of studies and design heterogeneity preclude causal inferences regarding comparative effectiveness. The effectiveness of CBT-based MHFA [29] may be partly attributable to its contextual specificity and crisis-oriented mechanisms aimed at psychological stabilization, which is consistent with reviews that characterize MHFA as a first-line psychological support during crises, rather than as a comprehensive long-term resilience curriculum [31,32].

The timing of intervention delivery may also have influenced resilience outcomes. In the post-COVID-19 period, one study reported improvements in resilience following positive psychotherapy and gratitude-based interventions [28], whereas a study conducted during the pandemic observed resilience enhancement associated with yoga and mindfulness-based interventions [25]. Although these temporal differences may suggest that distinct modalities operate through different mechanisms, the available data do not permit direct comparisons across recovery phases. Therefore, phase-specific interpretations should be considered provisional.

These findings suggest that intervention modalities may operate through different processes depending on the broader recovery context. During the acute phase of the COVID-19 pandemic, mindfulness-based and somatic interventions have been associated with gains in resilience, potentially through physiological stabilization and emotional regulation [33]. In contrast, in the post-pandemic period, interventions such as positive psychotherapy and gratitude-based programs have been associated with improvements in resilience, potentially through meaning-making and enhancement of positive affect [33]. However, these mechanistic explanations are theoretically grounded and were not directly tested in the included studies.

Taken together, despite heterogeneity in theoretical frameworks and intervention timing, the included programs were associated with improvements in nurses’ psychological outcomes, including emotional stabilization, reduction in PTSD symptoms, and, in some cases, enhanced resilience. Rather than establishing resilience as a definitively staged construct, the present findings are better interpreted as consistent with models that conceptualize resilience as dynamic and context-dependent.

In addition to theoretical and temporal considerations, the mode of delivery has emerged as a unifying feature across interventions. All included interventions were delivered through online platforms, either synchronously or asynchronously. Digital delivery may reduce logistical barriers to professional development in nursing, including irregular shift work, heavy workloads, and infection control constraints [16]. However, evidence regarding the effectiveness of online delivery formats remains mixed and inconclusive. A recent systematic review of 15 randomized controlled trials comparing online and face-to-face group-based psychosocial interventions reported generally comparable outcomes across formats, although the conclusions were limited by substantial heterogeneity [34]. Similarly, findings from online mindfulness-based interventions vary across outcomes and populations [35]. Consistent with this, the pooled effect size in the present review was statistically significant but small, suggesting that the delivery modality alone may be insufficient to ensure robust effects. The configuration of online modalities may also be relevant. Synchronous sessions enable real-time interaction and peer support, whereas asynchronous formats allow repeated engagement with core skills. Although the complementary use of these formats may enhance engagement, this possibility was not formally tested in the included studies and therefore warrants further empirical investigation. Collectively, these findings highlight the potential of digital resilience programs to address the complex psychological needs of nurses while underscoring the need for future interventions to move beyond delivery modality and focus on optimizing intervention intensity, participant engagement, and theoretical coherence to achieve more robust and sustained effects.

### Strengths and Limitations

This review has several strengths. To our knowledge, it is among the first systematic reviews and meta-analyses to examine resilience-enhancing programs for nurses in a post-pandemic context, an area of growing clinical and organizational importance. The study employed advanced analytical methods, including meta-analysis and meta-regression, to evaluate intervention effectiveness. It also highlights the increasing use of online delivery formats, reflecting broader digital transitions in healthcare education and support.

Despite these strengths, several limitations should be acknowledged. The number of eligible studies was relatively small, which may have limited statistical power, partly due to the inclusion of only English-language publications. This restriction may have led to the exclusion of relevant studies conducted in non-English-speaking contexts. Consequently, the precision and generalizability of the pooled estimates are limited and reflect the currently developing evidence base in this field rather than selective inclusion.

In addition, the included studies exhibited substantial heterogeneity. Although meta-regression analyses have examined several potential moderators, none have accounted for the observed between-study variability, suggesting the influence of unmeasured factors, such as differences in intervention intensity, participants’ baseline risk, stage of psychological recovery, or implementation fidelity. Particularly, although the interventions were described using different terms (e.g., mindfulness or trauma recovery), they were commonly included because they focused on measuring and enhancing resilience as an outcome. Therefore, variations in program components and delivery approaches may have contributed to the observed heterogeneity. The overall certainty of the evidence ranges from low to very low, warranting a cautious interpretation of the pooled estimates. Future research should include larger methodologically rigorous trials that examine the potential determinants and contextual factors influencing intervention effectiveness.

## 5. Conclusions

This systematic review and meta-analysis examined resilience-enhancing programs for nurses and evaluated their overall effectiveness. Based on six eligible studies, the random-effects meta-analysis demonstrated that these interventions had a statistically significant positive effect on nurses’ resilience, although substantial heterogeneity was observed across the studies. Resilience was defined as the primary outcome of this review and was the only quantitatively synthesized variable. Overall, these findings suggest that resilience-enhancing interventions may benefit nurses’ psychological well-being. However, further high-quality, adequately powered trials are needed to clarify the mechanisms, optimal delivery formats, and contextual factors associated with sustained effects. Nevertheless, resilience-enhancing programs may warrant consideration as one element of broader workforce mental health support efforts.

## Figures and Tables

**Table 1:** Grade Table for Studies Included.

First Author	Study Design	Risk of Bias	Inconsistency	Indirectness	Imprecision	Publication Bias	Overall Quality of Evidence
Henshall et al. [23]	Pilot RCT	Serious	Not serious	Not serious	Serious	Undetected	Low
Sis Çelik & Yarali [24]	RCT	Serious	Not serious	Not serious	Serious	Undetected	Low
Kim et al. [25]	RCT	Serious	Not serious	Not serious	Serious	Undetected	Low
Reyes et al. [26]	RCT	Serious	Not serious	Not serious	Serious	Undetected	Low
Dehghani [27]	Quasi-experimental	Serious	Not serious	Not serious	Serious	Undetected	Very low
Eweida et al. [28]	Quasi-experimental	Serious	Not serious	Not serious	Serious	Undetected	Very low

**Table 2:** Characteristics of Included Studies.

First Author	Year	Country	Design	Setting	Population				Outcome Variables
					N (Exp./Cont.)	Sampling	Inclusion Criteria	Recruitment	
Henshall et al. [24]	2023	UK	Pilot randomized trial	A mental health and community National Health Service (NHS) trust	107 (56/51)	Convenience sampling	Non-agency nurses of different levels of seniority, working across a wide range of clinical settings within the participating NHS trust	Online + Offline	Participant engagement; Acceptability of the REsOluTionN program; Resilience; Psychological well-being
Sis Çelik & Yarali [25]	2023	Turkey	Randomized controlled trial	Hospital	100 (50/50)	Snowball sampling	Inclusion Criteria: Nurses who had not undergone abdominal surgery in the previous three months; did not have uncontrolled hypertension; did not have glaucoma, hernias, or epilepsy; had not received a psychiatric diagnosis and treatment; had no diagnosed sleep disorders; had not previously practiced laughter yoga; and had actively worked during the pandemic.	Online	Resilience; Sleep quality
Kim et al. [26]	2024	Republic of Korea	Parallel randomized controlled trial	-	112 (56/56)	Convenience sampling	RNs who (1) were aged 23–40 years, (2) had access to the program via a computer or mobile device, (3) understood the purpose of the study and voluntarily consented to participate, (4) were not diagnosed with severe mental illness or taking medication for their treatment, and (5) self-scored lower than 80% (64 points) on the Korean version of the PTSD Checklist for DSM-5 (PCL-5)	Online	Functional health; Social support; Resilience; Post-traumatic stress; Depressive symptoms; Anxiety
Reyes et al. [27]	2024	USA	Randomized controlled trial	Clinical setting	60 (30/30)	Convenience sampling	Inclusion: (1) be at least 18 years of age; (2) be employed as a frontline nurse (RN or LPN) within the United States; (3) obtain a score of 33 or higher on the PTSD Checklist for DSM-5 (PCL-5), the established cutoff for a provisional PTSD diagnosis; and (4) possess an Android smartphone or iPhone (iOS 13 or later).	Online	PTSD symptom severity; Experiential avoidance; Rumination; Mindfulness; Resilience; Intervention satisfaction; Perceived usability of the app
Dehghani [28]	2025	Iran	A quasi-experimental study	Hospital	67 (39/38)	Convenience sampling	Nursing staff working in the coronavirus infection ward who had no psychological disorders and were willing to participate in the study.	Offline	Resilience
Eweida et al. [29]	2024	Saudi Arabia	A quasi-experimental study	Hospital	60 (30/30)	Convenience sampling	Nurses working in Intensive Care Units	Online	Resilience; Job insecurity; Organizational commitment; Turnover intention

**Table 3:** Characteristics of Resilience-Enhancing Programs for Nurses.

First Author	Program	Protocol	Theoretical Framework	Key Contents	Teaching Methods
Henshall et al. [24]	Resilience Enhancement Online Training for Nurses (REsOluTioN)	Over four weeks (10 h of structured content in addition to 4–8 h of flexible mentor meetings) (1) web-based 4 × 120 min large-group facilitated sessions on the weekly modules; (2) 4 × 30 min independent preparatory learning on the module topics prior to the large-group facilitated sessions; and (3) 8 small-group mentoring sessions delivered between 30 and 60 min at flexible timings, twice weekly (large-group facilitated sessions and mentor meetings were delivered via Teams).	None	(1) building hardiness and maintaining a positive outlook, (2) intellectual flexibility and emotional intelligence, (3) reflective and critical thinking, and (4) achieving life balance and enabling spirituality	Online (A blended synchronous and asynchronous learning approach) (group approach)
Sis Çelik & Yarali [25]	Laughter yoga sessions (mindfulness techniques, breathing exercises, etc.)	For four weeks (twice weekly) (via Zoom) 40 min/session (total 320 min) Three subgroups	None	Deep-breathing Exercises (5 min); Warm-up Exercises (10 min); Childish Games (10 min); Laughter Exercises (15 min)	Online (group approach)
Kim et al. [26]	Internet-Based Trauma Recovery Nursing Intervention Program	Eight sessions 30 min/session (total: 240 min) URL of each session was sent via a computer or mobile device (the researcher communicates with participants using a response letter)	Swanson’s theory of caring	Maintaining Belief; Being With; Knowing; Doing For; Enabling	Online (Individual approach)
Reyes et al. [27]	Mindfulness- and Acceptance-Based Smartphone App (Mindfulness technique)	For six weeks (daily mindfulness exercises and weekly learning materials)	ACT principles outlined by Hayes	Mindfulness meditation (daily audio-guided); Watching video and writing brief reflections (weekly)	Online (app, MetricWire, individual approach)
Dehghani [28]	Gratitude training program	For 28 days (daily)	None	Positive psychotherapy and gratitude	Online (WhatsApp, individual approach)
Eweida et al. [29]	Mental Health First Aid	For five weeks (twice weekly), 10 sessions One hour/session (total 600 min) Six subgroups (Zoom, webinar)	Cognitive–behavioral theory	RAPID (Rapport and reflective listening; Assessment; Prioritization; Intervention; Disposition and follow-up)	Online (group approach)

## Data Availability

No new data were generated in this study. Data were obtained from previously published studies.

## Supplementary Materials

The following supporting information can be downloaded at: https://www.mdpi.com/article/10.3390/healthcare14070906/s1, File S1: Detailed search strategy.
