# Supplementary material for: Resilience-Enhancing Programs for Nurses in the Era of COVID-19: A Systematic Review and Meta-Analysis
Source: Healthcare (Basel). 2026 Mar 31;14(7):906. doi: 10.3390/healthcare14070906 (PMC13073717; doi:10.3390/healthcare14070906)
Supplement: Supplementary file 1 [file healthcare-14-00906-s001.zip › healthcare-4119406-supplementary.pdf]

Appendix S1. Detailed search strategy

1. CINAHL

S#	Query	Results
S13	(S12 AND S11 AND S10 AND S1 AND S2)	6
S12	S7 OR S8 OR S9	185,910
S11	(S3 OR S4 OR S5 OR S6)	1,800,182
S10	Randomized Controlled Trial	262,971
S9	pandemic	111,132
S8	corona	2,119
S7	covid	170,858
S6	program	605,597
S5	intervention	630,112
S4	training	290,826
S3	education	820,221
S2	nurse	571,093
S1	resilience	24,073

2. Cochrane

S#	Query	Results
#15	#12 AND #3 AND #13 AND #14 AND #11	17
#14	#8 OR #9 OR #10	26,050
#13	#4 OR #5 OR #6 OR #7	789,686
#12	#1 OR #2	4,823
#11	Randomized Controlled Trial	1,161,816
#10	pandemic	9,472
#9	corona	1,361
#8	covid	23,563
#7	program	167,927
#6	intervention	632,032
#5	training	152,284
#4	education	122,052
#3	nurse	28,406
#2	Resilience, Psychological	2,367
#1	resilience	4,823

3. EMBASE

S#	Query	Results
#13	('resilience'/exp OR 'resilience') AND 'nurse' AND (randomized AND controlled AND trial) AND (education OR training OR intervention OR program) AND (corona OR covid OR pandemic)	23
#12	corona OR covid OR pandemic	616,266
#11	education OR training OR intervention OR program	6,070,966

#10	randomized AND controlled AND trial	1,511,892
#9	pandemic	347,416
#8	covid	511,957
#7	corona	32,479
#6	program	1,867,578
#5	intervention	1,631,593
#4	training	1,228,810
#3	education	2,649,522
#2	'nurse'	482,777
#1	'resilience'/exp OR 'resilience'	89,195

4. PubMed

S#	Query	Results
21	#14 AND #15 AND #16 AND #18 AND #20	34
20	#10 OR #11 OR #19	539,098
19	corona	22,579
18	#12 OR #13	881,616
16	#6 OR #7 OR #8 OR #9	14,509,671
15	#3 OR #4 OR #5	1,276,054
14	#1 OR #2	100,930
13	Randomized Controlled Trial[mesh]	189,902
12	Randomized Controlled Trial	881,616
11	pandemic	303,222
10	COVID	480,162
9	program	2,247,018
8	intervention	11,847,242
7	training	3,058,068
6	education	2,747,792
5	nursing[mesh]	270,524
4	nurses[mesh]	103,948
3	nurs*	1,268,122
2	Resilience, Psychological[mesh]	11,979
1	resilience	100,930
